# Surgical site infection and associated factors among women who underwent cesarean delivery at Gandhi Memorial Hospital, Addis Ababa, Ethiopia

**DOI:** 10.1186/s12884-026-08633-0

**Published:** 2026-01-07

**Authors:** Getinet Tilahun Simeneh, Dawit Tarko Alamenie, Soliyana Hailu Chekol, Tariku Deressa Abdana, Hailegebreal Kidane, Wubet Mihretu Workneh, Bisrat Tamene Bekele, Biniam Yohannes Wotango

**Affiliations:** 1 Health Service Quality and Patient Safety Directorate, Gandhi Memorial Hospital, Addis Ababa, Ethiopia; 2 Training and Research Directorate, Gandhi Memorial Hospital, Addis Ababa, Ethiopia; 3 Office of the Chief Executive Officer, Gandhi Memorial Hospital, Addis Ababa, Ethiopia; 4 Office of the Chief Clinical Director, Gandhi Memorial Hospital, Addis Ababa, Ethiopia; 5 Institute for Healthcare Improvement, Addis Ababa, Ethiopia; 6 Addis Ababa City Administration Health Bureau, Addis Ababa, Ethiopia

**Keywords:** Surgical site infection, Cesarean delivery, Women, Gandhi Memorial Hospital, Ethiopia

## Abstract

**Background:**

Surgical site infection continues to be among the most serious postoperative complications of cesarean delivery, leading to maternal morbidity and additional healthcare cost. Despite the rising trend of cesarean deliveries, evidence on the magnitude and risk factors of surgical site infection in local hospitals in Ethiopia remains limited. This study aimed to assess the magnitude and associated factors of surgical site infection among women who underwent cesarean delivery at Gandhi Memorial Hospital, Addis Ababa, Ethiopia.

**Methods:**

An institution-based retrospective cross-sectional study was conducted from 25 August to 15 September 2025 among women who underwent cesarean delivery at Gandhi Memorial Hospital Addis Ababa, Ethiopia between 1 May 2023 and 30 April 2025. A total of 485 medical records were selected using a systematic sampling technique. Data were collected from women’s medical records via a structured checklist and analyzed using Statistical Package for Social Science (SPSS) version 25. Descriptive statistics were used to summarize the data, and bivariable and multivariable logistic regression analyses were performed. Statistical significance was declared at a p- value < 0.05 with a 95% CI.

**Results:**

Among the reviewed records, 31 (6.4%; 95% CI: 4.49–8.36) women developed surgical site infection. Repeated digital vaginal examination (AOR = 2.44 (1.41, 5.19) increases the risk of bacterial introduction; delayed timing of prophylactic antibiotic (AOR = 2.32 (1.23, 4.29) reduces protective coverage at the time of incision; absence of vaginal cleansing right before surgery (AOR = 3.75 (1.26, 11.17) likely increases bacterial load and postoperative hemoglobin level < 11 g/dl (AOR = 5.16 (1.76, 11.19)) may reduce immune capacity. All were significantly associated with surgical site infection.

**Conclusion:**

This study found lower surgical site infection rates compared to previous Ethiopian studies; however, it remains a critical postoperative concern. Reducing frequent digital vaginal examinations, ensuring timely prophylactic antibiotics, promoting preoperative vaginal cleansing, and maintaining adequate maternal hemoglobin levels are critical to further reduce the risk of SSI. The retrospective nature of the study limits assessment of some factors, including operating room conditions.

## Background

Surgical site infection (SSI) is a healthcare-associated infection (HAI) and is defined as an infection occurring at the surgical site, particularly within 30 days following the procedure, or even within one year if prosthetic material is implanted [1]. SSI leads to significant patient morbidity, prolonged hospital stays, hospital readmissions, increased healthcare costs, and even, in some cases, death [2].

Cesarean delivery (CD) is one of the most frequently performed surgeries worldwide. While it is performed with indications, and helpful in reducing maternal and perinatal mortality rates, however, the procedure has its own set of complications, including SSI [3, 4]. Globally, the incidence of SSI following CD ranges from 3 to 15%, with significantly higher rates reported in developing countries [2, 5, 6]. In Ethiopia, hospital-based studies have documented SSI rates as high as 25%, underscoring a notable burden [7].

SSI following CD can be classified as superficial incisional, deep incisional, or organ/space infection [1, 2, 8]. It arises from numerous diverse factors that generally fall into three domains. Patient-specific factors include conditions such as diabetes mellitus, obesity, anemia, immunosuppression, and poor nutritional state. Procedure-specific factors such as prolonged operative time, poor skin preparation, and a lack of appropriate prophylactic antibiotics can affect the microbial environment at the surgical site. In addition, system-level factors, including poor sterilization control, the unavailability of trained personnel, and overcrowding further increase the likelihood of SSIs [1, 2, 9].

Women with SSI require extended hospitalization, repeated wound care, additional medications, and even reoperation, which then increases the cost of care and adds a significant consequence to the healthcare system [1, 8]. In addition, SSIs contribute to delay maternal recovery, cause chronic pain and emotional distress such as anxiety and depression. These psychological impacts may also hinder bonding with newborns, which then affecting both maternal and infant health. Collectively, these consequences shows critical public health concerns, as poor maternal mental health can influence family well-being and broader community health outcomes [9, 10].

According to the 2019 Ethiopian Mini Demographic and Health Survey (EMDHS) report, Ethiopia has significantly expanded maternal health services, leading to increased institutional deliveries and a rising rate of cesarean sections. However, evidence regarding adherence to quality and safe care, such as measures to prevent infections and the burden of SSI following cesarean delivery, remains limited. Although a few studies done in Ethiopia have reported SSI rates ranging from moderate to high, their findings are inconsistent and often context-specific, highlighting a persistent lack of comprehensive, facility-level data across the country [4, 6, 7, 11].

Gandhi Memorial Hospital (GMH) serves thousands of women each year and performs many cesarean deliveries [12]. However, the magnitude and associated factors of SSIs in this setting have not been assessed. This gap makes it difficult to develop evidence-based strategies for SSI intervention mechanisms. To improve post cesarean outcomes and enhance infection prevention systems, it is critical that maternity hospitals such as the GMH conduct local studies on SSIs and associated risk factors.

Therefore, the present study aimed to assess the magnitude and associated factors of surgical site infection among women who underwent cesarean delivery at Gandhi Memorial Hospital, Addis Ababa, Ethiopia, and providing evidence to inform clinical practice, hospital policy, and maternal health interventions.

## Methods

### Study setting and period

The study was conducted at GMH, a public hospital in Addis Ababa, Ethiopia. GMH specializes in maternal and neonatal health and provides maternal- and neonatal-related services. GMH serves as a teaching center for Addis Ababa University (AAU) students, including undergraduate medical students and residents specializing in obstetrics and gynecology. GMH is a referral hospital providing comprehensive maternal and neonatal care, performing a high volume of cesarean deliveries annually. In particular, the hospital performs many obstetric surgeries, mainly cesarean deliveries, with an annual average of 3,600 CD, constituting nearly half of all deliveries.

The study assessed the medical records of women who underwent cesarean delivery between May 1, 2023, and April 30, 2025, and data collection was conducted from 25 August to 15 September 2025.

### Study design

An institution-based retrospective cross-sectional chart review was carried out among women who underwent cesarean section at Gandhi Memorial Hospital, Addis Ababa, Ethiopia.

### Population

All women who gave birth via cesarean section composed the source population, and those who gave birth via cesarean section at Gandhi Memorial Hospital during the two- year period (from May 1, 2023, to April 30, 2025) composed the study population.

### Eligibility criteria

Women who underwent cesarean section at the GMH between May 1, 2023, and April 30, 2025, whose medical records contain complete data on preoperative, intraoperative and postoperative details, were included. However, records of women with incomplete data on key variables were excluded.

### Sample size determination

The required sample size for this study was calculated for both specific objectives i.e. magnitude and associated factors of surgical site infection, and the largest sample size obtained from the 1st objective, i.e., the proportion of SSIs: A single population proportion formula considering the assumption that the proportion of SSIs from the previous study was 11.6% [5]. With a 95% confidence level and considering the need for increased statistical power, the margin of error was 3% (d = 0.03). Thus, the sample size for the SSI was calculated as follows:

$$\text{Sample size} \left(\mathrm{n}\right)=\underline{Z_{a/2^2}\ast \mathrm{P} \left(1-P\right)/d^{2}}$$

Where p is the proportion of SSIs

q- Proportion of women who have no SSI

d- Margin of error.

Thus, $$\text{the sample size}\,\,{\left(\mathrm{n}\right)}=\underline{1.96^2\ast0.12\ast0.88/0.03^{2}=451}$$ 

Thus, the final sample size was 496 after adding a 10% nonresponse rate.

### Sampling procedure

There were 7200 cesarean deliveries at GMH between May 1, 2023, and April 30, 2025. To select the records of 496 women from the total number of cesarean deliveries, a systematic random sampling technique was used: N (the estimated number of cesarean deliveries in the two-year period in that hospital was 7200), and n (the required minimum sample size included a 10% nonresponse rate = 496), which yielded a sampling fraction (k): where k = N/n = > 7200/496 ≈ 14.

Therefore, every 14th medical record of a woman who underwent cesarean delivery between May 1, 2023, and April 30, 2025, was included. The first woman was selected randomly from the first 1–14 records via a lottery method, and every 14th woman was subsequently included in the study, starting from the woman who was selected.

### Operational definitions

#### Emergency cesarean section

A cesarean delivery is performed without prior scheduling, often due to either fetal or maternal conditions that necessitate immediate delivery [13].

#### Prolonged rupture of membranes

Defined as the rupture of membranes that lasts more than 12 hours before the onset of labor [14]. 

#### Repeated vaginal examination

If digital vaginal examinations are performed, five or more times, or if it is performed more frequently than every four hours during the course of labor [14, 15]. 

#### Surgical Site Infection (SSI)

If documentation indicates that a woman developed an infection involving the incision or deeper tissues within 30 days following a cesarean delivery, the infection is diagnosed and recorded on medical records of the woman [6, 16, 17].

### Data collection procedures

Data were collected via a structured and pretested data extraction checklist adapted from the literatures and national clinical guidelines for surgical care and infection prevention, with some modifications to the research objectives [1, 2, 6, 11]. The checklist contains socio-demographic data, obstetric and medical factors, and perioperative factors. Data were extracted from women’s charts from electronic medical records (EMRs). A total of 15 and 5 healthcare providers with sufficient knowledge and skills in data extraction were recruited as data collectors and supervisors, respectively. A daylong training session was given for data collectors and supervisors on the study objectives and data collection techniques.

### Data quality assurance

To ensure data quality, a pretest was employed on 5% of the sample size, i.e., 25 chart records at Zewuditu Memorial Hospital (ZMH), a public hospital in Addis Ababa. Data were collected by trained healthcare professionals under the supervision of assigned supervisors. All the checklists were reviewed for completeness and accuracy before, during, and after data collection. Additionally, the data were rechecked for completeness.

### Data processing and analysis

The collected data were exported to SPSS version 25 for analysis. Descriptive statistics such as frequencies, means, and standard deviations were employed to summarize the data, and the results are presented in texts, tables and graphs. The assumptions of logistic regression were checked before analysis. Bivariable logistic regression analysis was performed to assess the crude association between each independent variable and SSI. Variables with a p value < 0.25 in the bivariable analysis were candidates for the multivariable logistic regression model to identify significant factors of SSI. The fitness of the model was checked by the Hosmer and Lemeshow test at a P value > 0.05. Finally, the degree of association between variables was determined through adjusted odds ratios (AORs) with 95% confidence intervals (CIs) and p values less than 0.05, which were considered statistically significant.

## Results

### Maternal sociodemographic characteristics

A total of 496 women’s records were identified for review, and 485 charts were reviewed, for a review rate of 97.8%. Among all the reviewed charts, 281 (57.9%) were in the age group of 25–34 years, with a mean age of 28.36 years (± SD 4.746) and minimum and maximum ages of 18 and 41, respectively. A total of 442 (91.1%) of the study participants were married, and 419 (86.4%) women were permanent residents of Addis Ababa. With respect to the educational status and occupation of the study participants, nearly half of the 236 (48.7%) had attended secondary school and above, and more than half of the 268 (55.3%) women were private employees (Table 1).

**Table 1:** Sociodemographic characteristics of women who underwent Cesarean delivery at Gandhi memorial Hospital, addis Ababa, Ethiopia, from May 1, 2023, to April 30, 2025 (*n* = 485)

Background characteristics	Category	Frequency (n)	Percent (%)
Age of the mother	15–24 years	108	22.3
	25–34 years	281	57.9
	>=35 years	96	19.8
Marital status	Single	18	3.7
	Married	442	91.1
	Others ^a^	25	5.2
Residence	Addis Ababa	419	86.4
	Out of Addis Ababa	66	13.6
Educational status	No formal education	46	9.5
	Primary education	172	35.5
	Secondary and above	236	48.7
	Not mentioned	31	6.4
Occupation	House wife	74	15.3
	Private employee	268	55.3
	Governmental employee	110	22.7
	Others ^b^	33	6.8

Others ^a^*=*divorced, widowed, and not mentioned; others ^b^= student, daily laborer, and not mentioned

### Obstetric and medical-related characteristics

Among the total reviewed records, 461 (95.1%) women had ANC contact during their recent pregnancy, and more than half of the 259 (56.1%) of them had eight or more ANC contacts. More than two-thirds, 331 (68.2%), and more than three-fifths, 300 (61.9%), of the study participants were multigravida and multiparous, respectively. Among the reviewed records, 96 (19.8%) women had pregnancy-induced hypertension (PIH), and 44 (9.1%) had other comorbidities during their pregnancy period. The majority (466, 96.1%) of the women had singleton pregnancies. With respect to labor status and type of cesarean section, nearly half of the women (237, 48.9%) were not in labor and more than half (277, 57.1%) underwent emergency cesarean delivery. Among the study participants for whom a vaginal examination was indicated (*n* = 248), 109 (44%) underwent repeated vaginal examinations (Table 2).

**Table 2:** Obstetric and medical characteristics of women who underwent Cesarean delivery at Gandhi Memorial Hospital, Addis Ababa, Ethiopia, from May 1, 2023, to April 30, 2025 (*n* = 485)

Background characteristics	Category	Frequency (*n*)	Percent (%)
ANC contacts	Yes	461	95.1
	No	24	4.9
ANC contacts (*n* = 461)	< 4	42	9.1
	4–7	127	27.5
	≥ 8	259	56.2
	Contact number not mentioned	33	7.2
Gravidity	Primigravid	115	23.7
	Multigravid	331	68.2
	Grandmultigravid	39	8.0
Parity	Primipara	176	36.3
	Multipara	300	61.9
	Grandmultipara	9	1.9
PIH	Yes	96	19.8
	No	389	80.2
DM	Yes	29	6.0
	No	456	94.0
Chronic hypertension	Yes	11	2.3
	No	474	97.7
Anemia during pregnancy	Yes	35	7.2
	No	450	92.8
MUAC	< 23 cm	13	2.7
	≥ 23 cm	443	91.3
	Not recorded	29	5.9
Gestational age at delivery	Preterm	46	9.5
	Term	406	83.7
	post term	33	6.8
Type of pregnancy	Singleton	466	96.1
	Multiple	19	3.9
PROM	Yes	63	13.0
	No	422	87.0
MSFA	Yes	85	17.5
	No	400	82.5
Chorioamnionitis	Yes	6	1.2
	No	479	98.8
Labor status	Spontaneous	217	44.7
	Induced	31	6.4
	No labor	237	48.9
Frequency of vaginal examination	Normal	139	28.7
	Repeated	109	22.5
	Not done	237	48.9
Duration of labor (*n* = 248)	< 12 h	141	56.9
	≥ 12 h	107	43.1

### Perioperative-related characteristics

About six out of seven women, 413 (85.2%), who underwent cesarean delivery, had a preoperative hematocrit level of ≥ 33%. More than half, 277 (57.1%) of the operations were performed as emergencies, and four in five, 388 (80.0%) of the participants received prophylactic antibiotics within 60 min before the operation. With respect to the indications for cesarean delivery, about 31.1% of the procedures were performed due to multiple indications. More than half, 271 (55.9%) operations were performed by junior residents (R I–R II), with regional anesthesia used in almost all participants (99.0%) and skin preparation with two solutions applied in all participants. However, vaginal cleansing was performed for 368 (75.9%) women, and the safe surgery checklist was used for 92.0% of the women. Almost all (98.8%) procedures were performed via transverse (Pfannenstiel) incisions, and the majority (91.3%) of the procedures was completed within an hour. About 80.2% of the participants’ postoperative hemoglobin level was ≥ 11 g/dl. The length of postoperative hospital stay was evenly distributed, with nearly half (51.1%) of the women discharged in less than three days (Table 3).

**Table 3:** Perioperative-related characteristics of women who underwent Cesarean delivery at Gandhi Memorial Hospital, Addis Ababa, Ethiopia, from May 1, 2023, to April 30, 2025 (*n* = 485)

Background characteristics	Category	Frequency (*n*)	Percent (%)
Preoperative hematocrit	< 33%	72	14.8
	≥ 33%	413	85.2
Circumstances of operation	Emergency	277	57.1
	Elective	208	42.9
Antibiotic prophylaxis before surgery	Within 60 min before the surgery	388	80.0
	> 60 min before the surgery	63	13.0
	Not given	34	7.0
Indications of cesarean delivery	Previous cesarean scar related	120	24.7
	Non reassuring fetal heart rate	52	10.7
	Multiple indications	151	31.1
	LFSOL + GIII MSAF*	69	14.2
	Others ^c^	93	19.2
Surgeon’s level (in years)	R I or R II	271	55.9
	R III or R IV	193	39.8
	Consultant	21	4.3
Types of anesthesia	Regional	480	99.0
	General	5	1.0
Skin preparation before surgery	With two solutions	485	100
Vaginal cleansing done right before surgery	Yes	368	75.9
	No	117	24.1
Safe surgery checklist used	Yes	446	92.0
	No	39	8.0
Type of Abdominal incision	Vertical (Midline)	6	1.2
	Transverse (Pfannenstiel)	479	98.8
Duration of surgery	≤ 1 h	443	91.3
	> 1 h	42	8.7
New-born status at time of delivery	Alive	482	99.4
	Stillbirth	3	0.6
Estimated blood loss	≤ 500 ml	473	97.5
	> 500 ml	12	2.5
Postoperative hemoglobin	< 11 g/dl	96	19.8
	≥ 11 g/dl	389	80.2
Postoperative hospital stays	< 3days	248	51.1
	≥ 3 days	237	48.9

LFSOL + GIII MSAF* Latent first stage of labor with grade three meconium-stained amniotic fluid,*R I*,* RII*,* RIII and RIV* Year one, year two, year three and year four residents, respectively, others^c^mal presentation, multiple pregnancy, failed induction, placenta previa, macrosomia, oligohydramnios, and IUGR

### Magnitude of surgical site infection

**Fig. 1:**
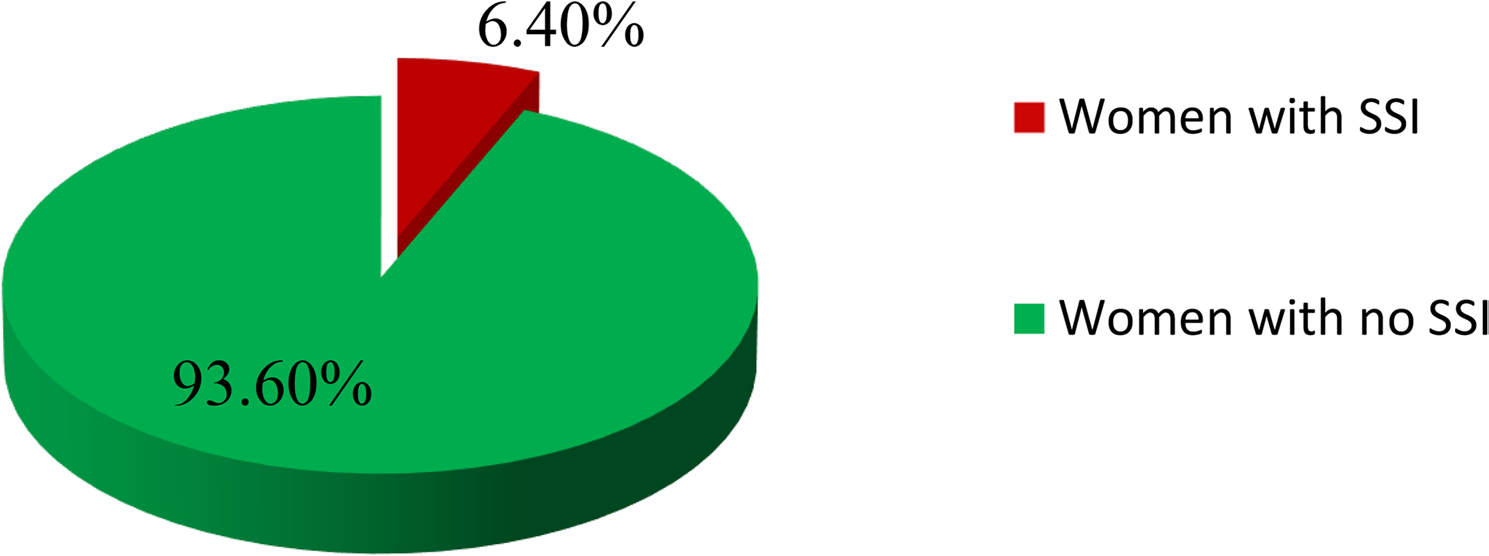
Magnitude of surgical site infection among women who underwent cesarean delivery at Gandhi Memorial Hospital, Addis Ababa, Ethiopia, from May 1, 2023, to April 30, 2025 (*n*=485)

Among the 485 women who underwent cesarean delivery, 31 (6.4%; 95% CI: 4.49–8.36) developed surgical site infections. Among these, 16 of them had superficial incisional infections, 13 had deep incisional infections, and 2 had organ/space infections (Fig. 1).

### Factors associated with SSI

In the bivariable analysis, anemia during pregnancy, repeated digital vaginal examination, PROM, MSAF, timing of antibiotic prophylaxis, vaginal cleansing, postoperative hospital stay and postoperative hemoglobin level were associated with SSI at a p value of less than 0.25. These 8 explanatory variables with P values < 0.25 in the bivariable logistic regression analysis were subsequently regressed against the SSI. As a result, in the multivariable logistic regression analysis, repeated digital vaginal examination, antibiotic prophylaxis before surgery, vaginal cleansing, and postoperative hemoglobin were significantly associated with SSI at a P value of < 0.05.

Accordingly, women who underwent repeated digital vaginal examinations were more than twice as likely to develop SSI than those whose examinations were normal [AOR = 2.44 (1.41, 5.19); *p* = 0.002]. Similarly, the odds of developing SSI were more than two and four times greater among women who received prophylactic antibiotics more than one hour before surgery and those who did not receive antibiotics, respectively, than among women who received antibiotics within one hour before surgery [AOR = 2.32 (1.23, 4.29), *p* = 0.001] and [AOR = 4.31 (1.56, 7.68), *p* = 0.043]. Compared with their counterparts, women who did not undergo vaginal cleansing just before surgery had a 4-fold greater risk of developing SSIs [AOR = 3.75 (1.26, 11.17); *p* = 0.018], whereas the odds of having SSIs were 5.16 times [AOR = 5.16 (1.76, 11.19), *p* = 0.003] greater among women with postoperative hemoglobin levels < 11 g/dl (Table 4).

**Table 4:** Factors associated with surgical site infection among women who underwent Cesarean delivery at Gandhi Memorial Hospital, Addis Ababa, Ethiopia, from May 1, 2023, to April 30, 2025 (*n* = 485)

Variables		SSI		COR with 95% CI	AOR with 95% CI
		Yes N (%)	No N (%)		
Anemia during pregnancy	Yes	7 (20.0)	28 (80.0)	4.44 (1.76, 11.19)	0.28 (0.07, 1.01)
	No	24 (5.3)	426 (94.7)	1	1
Vaginal examination	Normal	7 (5.0)	132 (95.0)	1	1
	Repeated	14 (12.8)	95 (87.2)	2.78 (1.23, 6.75)	2.44 (1.41, 5.19)^*^
	Not done	10 (4.2)	227 (95.8)	0.83 (0.24, 1.97)	1.25 (0.06, 8.64)
PROM	Yes	8 (12.7)	55 (87.3)	2.52 (1.08, 5.92)	1.31 (0.43, 4.01)
	No	23 (5.5)	399 (94.5)	1	1
MSAF	Yes	11 (12.9)	74 (87.1)	2.82 (1.29, 6.14)	1.38 (0.50, 3.78)
	No	20 (5.0)	380 (95.0)	1	1
Antibiotic prophylaxis	Within 60 min before surgery	18 (4.6)	370 (95.4)	1	1
	More than 60 min before surgery	7 (11.1)	56 (88.9)	2.57 (1.23, 6.64)	2.32 (1.23, 4.29)*
	Not given	6 (17.6)	28 (82.4)	4.40 (2.21, 10.75)	4.31 (1.56, 7.68)^*^
Vaginal cleansing just before surgery	Yes	20 (5.4)	348 (94.6)	1	1
	No	11 (9.4)	106 (90.6)	1.81 (0.84, 3.89)	3.75 (1.26, 11.17)*
Postoperative hospital stays	< 3 days	12 (4.8)	236 (95.2)	1	1
	≥ 3 days	19 (8.0)	218 (92.0)	1.71 (0.81, 3.61)	0.52 (0.18, 1.53)
Postoperative hemoglobin level	< 11 g/dl	15 (15.6)	81 (84.4)	4.32 (2.05, 9.09)	5.16 (1.76, 11.19)^*^
	≥ 11 g/dl	16 (4.1)	373 (95.9)	1	1

1, Reference; *Statistically significant at *p *<0.05

## Discussion

This study aimed to assess the magnitude of surgical site infection and identify associated factors among women who underwent cesarean delivery in the study setting. The magnitude of SSI was 6.4%, with a 95% CI of 4.49–8.36, and repeated digital vaginal examination, the timing of antibiotic prophylaxis before surgery, vaginal cleansing, and postoperative hemoglobin were significantly associated with SSI. This study was similar to reports from other Ethiopian hospitals, such as Lemlem Karl Hospital Maichew (6.8%), Debre Tabor (8%) and Felegehiwot referral hospitals (7.8%) [6, 18, 19]. This study is also comparable with previous studies conducted in Saudi Arabia (4.7%) and Uganda (7.9%) [20, 21].

However, the magnitude of SSI in the current study was greater than that reported in developed countries such as the United States (3.0%) and Denmark (3.2%) [22, 23]. This finding is also greater than those of studies reported in African countries such as South Africa (2.9%), Rwanda (3.5%) and Kenya (2.1%) [24–26]. This difference might be due to differences in healthcare infrastructure, resources and postoperative follow-up systems. In high-income settings, a continuous infection surveillance system and strict adherence to surgical checklists might markedly reduce the incidence of SSI, while countries with limited resources, including Ethiopia, still face challenges related to infection prevention mechanisms. Unlike the current study settings, which often face challenges such as overcrowded wards, inconsistent antibiotic timing, and repeated digital vaginal examinations, which reflect modifiable clinical practices and maternal vulnerability that increase infection risk, the majority of the studies mentioned above might involve continuous staff training and stronger monitoring and accountability systems that support lower SSI rates.

Conversely, the findings of the present study are lower than those reported from Kosovo (9.9%), Tanzania (10.9%), and systematic reviews and meta-analyses in Africa (11%) [27–29]. The findings of the current study are also lower than those of studies performed in other hospitals in Ethiopia, such as public hospitals in Harar city (12.3%), Jimma University Medical Centre (19.7%), Hawassa University Comprehensive Specialized Hospital (11.8%), Debre Markos Referral Hospital (25.4%), and a systematic review and meta-analysis performed in Ethiopia (12.3%) [4, 7, 11, 30, 31]. The lower SSI rate in this study might be attributed to the fact that it was performed in a specialized maternity hospital in Addis Ababa, where access to skilled professionals, improved surgical techniques and improved infection prevention practices might have contributed to the lower SSI rate. The relatively lower magnitude of the SSI might also be attributed to differences in study design and data collection methods. For instance, some of the previous studies employed prospective designs with longer post discharge follow-up periods, thereby yielding higher SSI rates.

Regarding the factors associated with SSI, the current study identified that women who underwent repeated vaginal examinations during their labor course were more than twice as likely to develop SSI as those who had a normal frequency of examinations. This finding is supported by studies from Tanzania [28], Debre Markos Referral Hospital [7] and Addis Ababa [5], which reported a strong association between frequent vaginal examinations and postoperative infection. This association could be due to the increased risk of ascending bacterial contamination from the lower genital tract into the uterine cavity during repeated digital vaginal examinations.

Antibiotic prophylaxis before surgery is another important factor for SSI. Women who received prophylactic antibiotics more than one hour before surgery and those who did not receive prophylactic antibiotics before surgery were more than 2 and 4 times more likely to develop SSI than those who received antibiotics within one hour prior to incision, respectively. This association is consistent with studies conducted in Kenya [25] and Jimma University Medical Centre [30]. The likely explanation is that antibiotic administration within the recommended time before surgery ensures optimal serum and tissue concentrations at the time of surgical exposure and can reduce microbial contamination and infection risk. In contrast, when antibiotics are administered too early (an hour before incision) or not at all, bacteria can contaminate the wound and increase the risk of surgical site infection.

The study also revealed that women who did not undergo vaginal cleansing just before the incision were four times more likely to develop SSI than their counterparts. Other studies have shown that vaginal cleansing via antiseptic agents before cesarean delivery can decrease the bacterial load in the lower genital tract and reduce the risk of ascending infection and postoperative surgical site contamination [2].

Furthermore, the findings of the present study revealed that low postoperative hemoglobin levels were significantly associated with SSI. Women with a postoperative Hb level < 11 g/dl had a fivefold greater risk of developing SSI than those with a Hb level ≥ 11 g/dl. A study at Debretabor General Hospital reported this association [6], indicating that women with postoperative anemia were more likely to develop SSI. This is the fact that low hemoglobin impairs tissue oxygenation and immune function and delays wound healing, creating favorable conditions for bacterial growth and infection.

### Limitations of the study

This study is limited by its retrospective nature, which relies on secondary data; as a result, environmental factors such as operating room conditions were not assessed. However, the use of existing records of women allowed for the efficient utilization of available information and enabled valuable insight into infection patterns and associated factors within the study setting.

## Conclusion

The findings of the present study revealed that about 1 in 16 women who underwent cesarean delivery developed surgical site infection. Repeated digital vaginal examinations, delayed timing of antibiotic prophylaxis, absence of vaginal cleansing and postoperative hemoglobin level < g/dl were significantly associated with SSI. The findings highlight the need to strengthen initiatives aimed at reducing SSI by improving service quality care and addressing all potential risk factors. Hospitals should adhere to aseptic techniques not only during cesarean delivery but also during the course of labor, including minimizing frequent digital vaginal examinations to reduce the incidence of ascending infections. Healthcare providers working in the area must ensure the timely administration of prophylactic antibiotics to all women before incision. To reduce bacterial contamination, routine vaginal cleansing with antiseptic solutions before cesarean delivery should be integrated into standard obstetric protocols. Efforts should be made to prevent and correct maternal anemia by counseling women on nutrition, iron supplementation and strict monitoring during both the antenatal and postoperative periods to improve wound healing and reduce the risk of infection.

## Data Availability

The datasets generated and/or analyzed during the current study are not publicly available owing to ethical restrictions of protecting the privacy and confidentiality of the study subjects but are available from the corresponding author upon reasonable request.

## Abbreviations

AOR: Adjusted odds ratio
CD: Caesarean delivery
CI: Confident interval
GMH: Gandhi Memorial Hospital
HAI: Healthcare-associated infection
HGB: Hemoglobin
SSI: Surgical site infection
